# Microfluidic Biosensor Array with Integrated Poly(2,7-Carbazole)/Fullerene-Based Photodiodes for Rapid Multiplexed Detection of Pathogens

**DOI:** 10.3390/s131215898

**Published:** 2013-11-25

**Authors:** Nuno Miguel Matos Pires, Tao Dong

**Affiliations:** Department of Micro and Nano Systems Technology, Faculty of Technology and Maritime Sciences, Vestfold University College, Box 2243, N-3103 Tonsberg, Norway; E-Mail: nuno.pires@hive.no

**Keywords:** organic photodetector, microfluidic integration, multiplexed detection, environmental monitoring, point-of-care

## Abstract

A multiplexed microfluidic biosensor made of poly(methylmethacrylate) (PMMA) was integrated into an array of organic blend heterojunction photodiodes (OPDs) for chemiluminescent detection of pathogens. Waterborne *Escherichia coli* O157:H7, *Campylobacter jejuni* and adenovirus were targeted in the PMMA chip, and detection of captured pathogens was conducted by poly(2,7-carbazole)/fullerene OPDs which showed a responsivity over 0.20 A/W at 425 nm. The limits of chemiluminescent detection were 5 × 10^5^ cells/mL for *E. coli*, 1 × 10^5^ cells/mL for *C. jejuni*, and 1 × 10^−8^ mg/mL for adenovirus. Parallel analysis for all three analytes in less than 35 min was demonstrated. Further recovery tests illustrated the potential of the integrated biosensor for detecting bacteria in real water samples.

## 1. Introduction

Organic thin film photodiodes (OPDs) hold great promise as integrated optical sensors in lab-on-a-chip devices [1,2]. These sensors combine the simplicity and low-cost of spin-coating, inkjet printing or spray-coating fabrication techniques with the ready availability of glass or flexible plastic substrates [3–5]. Thus, the OPD technology offers great potential for developing simple, inexpensive, portable, disposable sensor devices for use in the field. Recently, the use of OPDs in integrated microfluidic systems for both fluorescence and chemiluminescence detection of analytes has been reported [6–9]. A fluorescence detector for biomarkers and molecular probes [7,9] was constructed from heterojunction photodiodes of copper phthalocyanine (CuPc) and fullerene (C_60_), whilst chemiluminescence sensors [4,6] were developed using blend heterojunctions of poly(3-hexylthiophene) (P3HT) and [6,6]-phenyl-C_61_-butyric acid methyl ester (PC_60_BM). However, the insufficient sensitivity and high detection limit (LOD) of the reported detection methods may limit the use of OPD-based analytical systems compared to conventional analytical chips.

In the past few years there has been tremendous progress in the field of OPD technology, and new conjugated polymers and C_70_ derivatives have been developed with the purpose of improving the power conversion efficiency of organic solar cells [10,11]. Poly[N-9′-heptadecanyl-2,7-carbazole-alt-5,5-(4′,7′-di-2-thienyl-2′,1′,3′-benzothiadiazole)] (PCDTBT) is one of new conjugated polymers that forms a blend heterojunction with PC_70_BM, resulting in detectors with enhanced light absorption, higher short-circuit current density and lower dark current compared to P3HT:PC_60_BM blends [12]. Owing to these unique characteristics, the PCDTBT:PC_70_BM heterojunction photodiode may be promising for OPD-based chemical and biological sensors in applications requiring high detection sensitivities. Detection of pathogens in water, for instance, often involves analysis for low concentrations of protozoa, bacteria and viruses in the samples. Highly sensitive methods are thus required for monitoring the safety of water [13–15], and the tests are preferred to be conducted at the point of care in order to obtain rapid results [16]. An ideal point-of-care device should perform multiplexed tests as both drinking water and surface water are vulnerable to *Escherichia coli*, *Campylobacter* and adenovirus, among others [17,18]. Although arrays of organic light emitting diodes have been used for multiplexed detection [19], no OPD-based multi-analyte sensor has yet been demonstrated.

Multiplexed detection of pathogens can be accomplished using immunological methods [20,21]. Immunoassays are extensively employed in lab-on-a-chip devices, and rapid analyte detection is typically achieved by use of either optical or electrochemical methods [22–24]. The electrochemical method is discouraged in disposable microfluidic chips as it is often affected by variations of temperature, pH and ionic concentrations, whilst the development of optical methods for microfluidic sensors has recently received considerable attention in on-site sensing [2]. Thus, a number of optical sensors, such as silicon photodiode arrays, ring resonators and photonic crystals have been studied in integrated microfluidic systems [25,26]. These integrated detectors involve complex, high-cost fabrication which limits their feasibility for point-of-use testing despite their high sensitivity and potential for multiplexed detection. Instead, the low-cost fabrication of OPDs may facilitate multiplexing of the integrated devices and allows their mass production.

This work presents a multiplexed optical-biosensor platform with highly sensitive organic photodiodes integrated to a single microfluidic chip. Detection of *E. coli*, *Campylobacter jejuni* and adenovirus was conducted by integrated PCDTBT:PC_70_BM blend heterojunction photodiodes. Immunoassays were performed on functionalized plastic microfluidic substrate and the luminol chemiluminescence reaction was used as the transduction mechanism. Chemiluminescence may be an ideal solution to on-site applications comparing to other optical readout methods [27], as it allows great reduction in the complexity of the detection system design [28]. The optoelectronic performance of a single PCDTBT:PC_70_BM sensor pixel was characterized before analysing the pathogen detection. Individual and multiplexed detection of waterborne pathogens with the OPD-integrated microfluidic platform was then demonstrated in both artificial and real water samples.

## 2. Experimental Section

### 2.1. Integrated System Design and Fabrication

**Figure 1.**
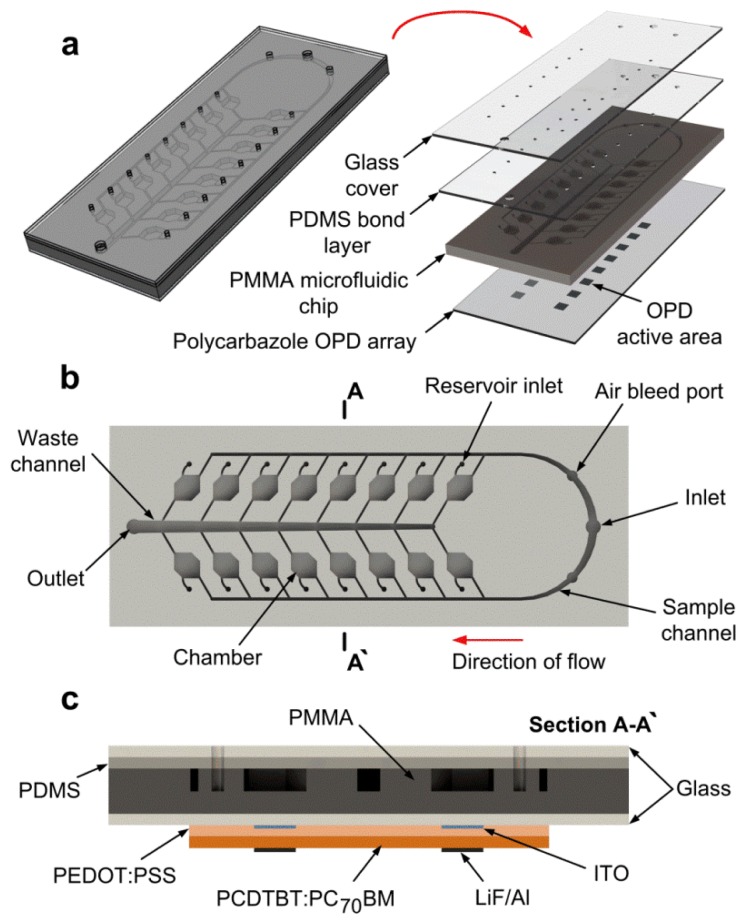
(**a**) Illustration of the multiplexed optical-biosensor platform integrating an array of polycarbazole OPDs to a hybrid microfluidic chip made of PMMA and PDMS. (**b**) Top view of the PMMA microfluidic substrate with ∼30 mm^3^ volume chambers. (**c**) Cross-section view of the integrated device illustrating all device components (not to scale).

The multiplexed optical-biosensor platform is depicted in Figure 1. It is mainly composed of an integrated array of sixteen OPDs and a hybrid microfluidic chip of poly(methyl methacrylate) (PMMA) and poly(dimethylsiloxane) (PDMS).

Microfluidic structures on PMMA with high feature resolution can be easily replicated by injection moulding or hot embossing processes while substrates of PDMS involve simple and robust bonding at low temperature [29]. The channel networks on PMMA were constructed resembling the angio-architecture [24,30]. The sample is loaded in the microfluidic chip through only one inlet. The fluid is then guided along two microchannels and enters ∼30 mm^3^ volume chambers, where the chemiluminescence assays are performed onto antibody functionalized PMMA surfaces. Further, the light generated from the chemiluminescent reactions is detected by 16 mm^2^ active area OPDs aligned below the chambers. The minimum distance between adjacent OPDs in the array is ∼4 mm.

PMMA was selected as the microfluidic substrate for the assays due to its outstanding transparency in the visible range, making it a good candidate for the chemiluminescence detection. Furthermore, the hybrid chip of PMMA and PDMS allows the realization of microchannels with high conformity, enabling complete filling of reagents within the channels networks. The PDMS-PMMA interface enables the fabrication of microfluidic integrated valves or pumps for autonomous devices. In addition, large-area bonding of microfluidic chips, ideal to multiplexed detection devices, can be accomplished using the PDMS-PMMA interface [31].

The OPDs employed in this study were PCDTBT:PC_70_BM heterojunction photodiodes. The enhanced performance of the PCDTBT:PC_70_BM photodiode pixel was previously described [32]. To achieve high analytical sensitivity, the pixel was designed with 120-nm-thick PCDTBT:PC_70_BM active layer and 40-nm-thick poly(3,4-ethylenedioxythiophene):polystyrene sulfonate (PEDOT:PSS) hole transport layer. Furthermore, the remarkable stability of the structure of PCDTBT and its large ionization potential makes the PCDTBT-based photodetector highly stable against ambient conditions of temperature, oxygen and/or humidity [12]. This would make the PCDTBT:PC_70_BM device interesting for point-of-care applications.

The PMMA microfluidic chip was realized by an injection moulding process, to which were used Ni-based master moulds. An UV-LIGA technique was employed for the mould fabrication. Ni mould disks were pre-coated by thick SU-8 photoresist, and then Ni-electroplating was conducted. The chip replication was performed by a standard injection moulding machine using high packing pressure (>100 MPa). As with our previous similar chip fabrication, the channel network [33,34] connecting sixteen chambers was optimized using FEM. After replication, the PMMA plate was cleaned with ethyl alcohol and pre-treated with oxygen plasma. The plate was then exposed to a 1% (v/v) solution of (3-aminopropyl)triethoxysilane (3-APTES, purchased from Sigma Aldrich, St. Louis, MO, USA) in deionized water (DI) for 20 min; whilst the PDMS substrate was treated with oxygen plasma. After washing with DI water, the silanized PMMA was irreversibly bonded to the plasma activated PDMS at room temperature. Prior to bonding, the fluidic chambers in the PMMA was subjected to 50 nM *N*-hydroxysulfosuccinimide (NHS) and 200 mM *N*-ethyl-*N*′-(3-dimethylaminopropyl)-carbodiimide hydrochloride (EDC) for 30 min at room temperature. The method for PMMA-PDMS bonding is very stable and can withstand fluid flow pressures above 500 kPa [31]. The microfluidic setup was completed by attaching the PDMS to a glass slide serving as the chip-to-world interface. The holes for the inlets and outlet of the multiplexed optical-biosensor platform (see Figure 1) were previously punched through the PDMS. Further, the PMMA was attached to the array of OPDs prepared on commercial ITO-coated (100 nm) glass substrate. This substrate has been patterned using HCl/HNO_3_ wet etching to form sixteen devices. After sequential washing with DI water, acetone and isopropanol and treatment with UV-ozone, the etched ITO electrodes were coated by the 40-nm PEDOT:PSS film. The 120-nm PCDTBT:PC_70_BM was spin-coated on top of the PEDOT:PSS from 1:4 blend mixture of PCDTBT and PC_70_BM in chloroform. The content was then dried at 60 °C under nitrogen for 1 h. Subsequently, the LiF/Al electrodes (∼100 nm) were deposited on top of the PCDTBT:PC_70_BM in a vacuum of 3 × 10^−4^ Pa.

### 2.2. Characterization of OPD Pixel Performance

The current-voltage (*J*-*V*) characteristics of the PCDTBT:PC_70_BM pixel were analysed illuminating the pixel with a YLLD blue laser diode (*λ* = 405 nm, Shanghai Yuli Technology Co., Ltd., Shanghai, China). The 405-nm diode was used since light emissions of horseradish peroxidase-luminol-peroxide reactions, typically employed in chemiluminescent sensors, present a maximum at 425 nm [27]. *J*-*V* curves were obtained using a 2600A source measure unit (SMU, Keithley Instruments Inc., Cleveland, OH, USA). The responsivity (*R*) of the PCDTBT:PC_70_BM photodetector was obtained in terms of external quantum efficiency using a 150 W xenon lamp, an Omni-λ monochromator (Zolix Instruments Co. Ltd., Beijing, China), a Keithley 2600A SMU, and a calibrated silicon photodiode (Hamamatsu Photonics K.K., Hamamatsu, Japan). *R* was thus determined from the following relation [35]:

(1) $$R=\frac{\eta \lambda q}{hc}$$

where *η* is the external quantum efficiency, *λ* represents the incident light wavelength, *q* denotes the elementary charge constant, *h* is the Planck constant and *c* is the light velocity.

### 2.3. Multiplexed Pathogen Detection

**Figure 2.**
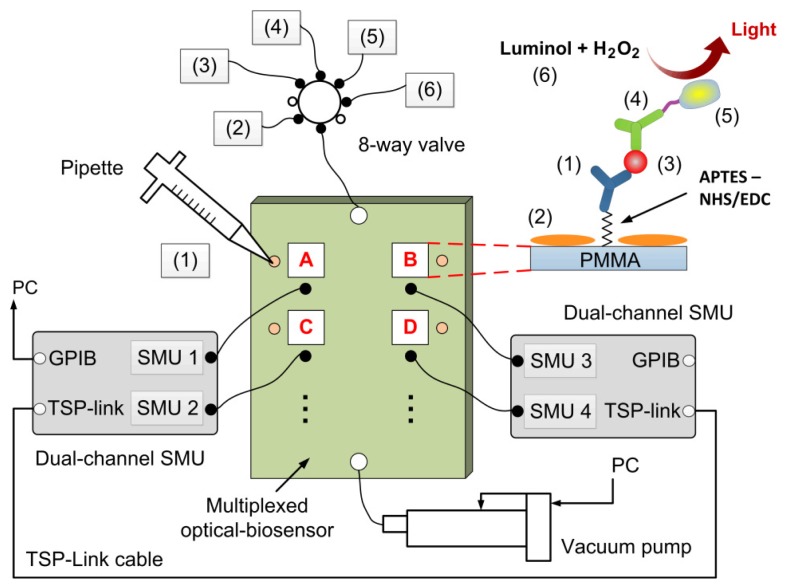
Schematic diagram of the experimental setup for the multiplexed pathogen detection. Four detection zones (A–D, chamber + OPD) were monitored using two multi-channel SMUs, and chemiluminescent immunoassays were performed following the protocol: (1) monoclonal antibody; (2) blocking buffer; (3) pathogen-spiked sample; (4) biotinylated antibody; (5) streptavidin-HRP conjugate; (6) chemiluminescent substrate.

The experiments for the multiplexed pathogen detection were conducted with the setup illustrated in Figure 2. A vacuum pump was used to suck the fluid into the multiplexed optical-biosensor platform while an eight-way valve system was employed to control the delivery of reagents. The inlet and outlet of the platform was connected to the valve and pump respectively via PEEK tubing (508 μm inner diameter; 1,587.5 μm outer diameter). For tubing connections, four NanoPort™ assemblies (N-333, Upchurch Scientific, Oak Harbor, WA USA) were attached to the holes for the inlet, outlet and air-bleeders (see Figure 1). Additionally, 80 μL volume NanoPort™ reservoirs (N-131, Upchurch Scientific) were added to the reservoir inlets of the platform, used for surface treatment.

The multiplexed detection was performed by targeting heat-killed *E. coli* O157:H7 cells, heat-killed *Campylobacter jejuni* cells and inactivated adenovirus antigen in three chambers of the platform (A, B and C in Figure 2). The overall assay procedure for detection of inactivated bacteria and virus is summarized below. All spiked samples and buffers were prepared in water produced by a Milli-Q^®^ system (Millipore, Milford, MA USA). Each chamber was coated with monoclonal antibody specific to an organism. The antibody solutions (10 μg/mL) were pipetted onto reservoir inlets of chambers A–C, and driven into the chip by the vacuum pump. The content was incubated for 2 h within the chambers, allowing covalent binding of antibody to APTES-activated PMMA surface via NHS/EDC chemistry [36]. No antibody was coated in chamber D which acted as a negative control. StartingBlock™ buffer in phosphate-buffered saline (PBS, pH 7.4) was further used to block the chamber surface. After the chip was flushed with PBS, 180 μL pathogen-spiked samples was loaded into chip via the inlet and incubated for 15 min within the chambers. This was followed by addition of 1 μg/mL biotinylated antibodies. After PBS flushing the chip was incubated with 0.015 μg/mL streptavidin horseradish peroxidase (HRP) conjugate. Following an additional flushing step, a 1:1 mixture of SuperSignal® chemiluminescence reagents (180 μL) was added to the chip in order to obtain the chemiluminescence signal. Gas bubbles generated during each assay step have been removed through the air-bleeders [32].

The chemiluminescence signal (∼425 nm) was measured as photogenerated current by the OPDs. To detect chemiluminescence in chambers A to D, four organic photodetectors of sixteen devices fabricated in the array were used. The signals obtained from each chamber were recorded at 0–10 min after the addition of the SuperSignal® reagents. Parallel measurements of photocurrent from four OPDs were ensured by two Keithley 2600A SMUs, each one equipped with two source-measure units and an embedded Test Script Processor (TSP). Both instruments were interconnected via TSP-Link, and obtained data was transferred to a PC by the master SMU via GPIB interface.

## 3. Results and Discussion

### 3.1. Characterization of Optoelectronic Performance

**Figure 3.**
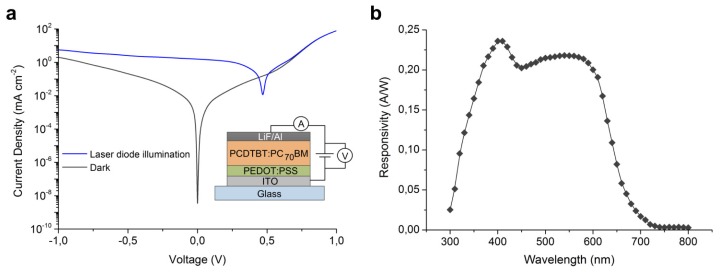
(**a**) *J*-*V* curves for the OPD pixel in dark and under laser diode illumination. Inset shows the ITO/PEDOT:PSS/PCDTBT:PC_70_BM/LiF/Al architecture of the OPD pixel. (**b**) Photosensitivity spectral response for the OPD pixel.

The optoelectronic performance of the OPD was characterized prior to integration to the PMMA-PDMS hybrid chip. Figure 3a shows the *J*-*V* curves of the photodetector in the dark and under blue laser diode illumination. The curves present an asymmetric behaviour which indicates an effective collection of photoinduced charge carriers [35]. The dark current density (*J*_dark_) under 0.1 V reverse bias was 9.55 × 10^−3^ mA/cm^2^. This was significantly higher than the dark current of 2.88 × 10^−9^ mA/cm^2^ measured at 0 V. Furthermore, *R* was estimated for the wavelength range of 300 to 800 nm (Figure 3b). For 425 nm, *R* was 0.21 A/W which contrasts with a response of 0.10 A/W, at same wavelength, obtained by an OPD pixel prepared from P3HT:PC_60_BM blend heterojunction [8]. A response of 0.24 A/W was found for 405 nm, and a second method to determine *R* was used for comparison. In that second method the photocurrent was plotted against variably controlled light intensity of the 405-nm laser diode. From the slope of that plot, *R* was calculated to be 0.22 A/W, thus agreeing well with the first method. Moreover, both *J*_dark_ and *R* did not differ significantly between ten pixels of sixteen fabricated in the OPD array. The maximum difference was 8% and 29% for *R* and *J*_dark_ respectively.

### 3.2. Chemiluminescence Detection on Silanized PMMA Surface

**Figure 4.**
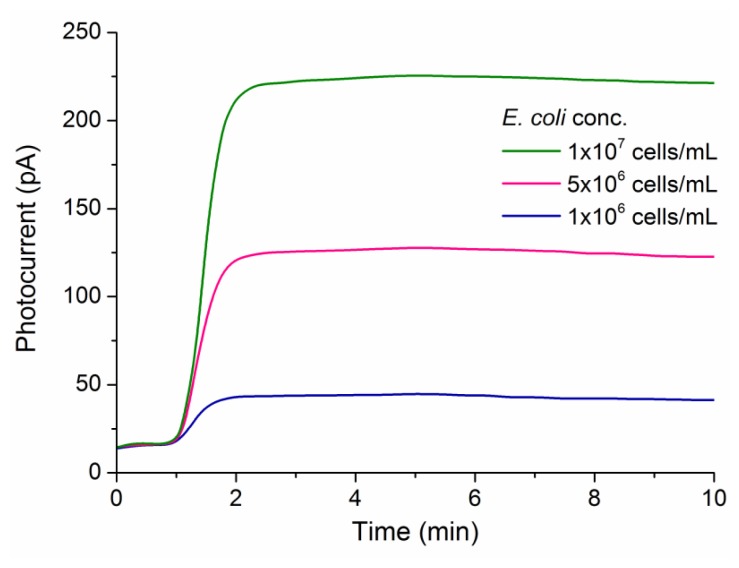
Transient chemiluminescence signals for detection of *E. coli* O157:H7 cells with one PCDTBT:PC_70_BM OPD pixel.

The binding of pathogen to antibody-coupled silanized PMMA surface generated light through the reaction of the chemiluminescence reagents by the HRP conjugates. The light intensity measured by the OPD pixels varies with the density of pathogen at the PMMA surface. This is demonstrated by the transient photocurrent responses plotted in Figure 4. Measurements were performed applying no bias voltage (Figure 3a) for all pathogen detection tests. Baseline readings were taken at 0 s to 60 s after the addition of the chemiluminescence reagents. The baseline varied between 15 pA and 22 pA, which was higher than the dark current previously measured. This may have occurred due to the presence of some stray light during measurements. Although the potential of the pixel to detect analytes in 1–2 min (starting point for the signal plateau), the region of analysis was defined at the 3–7 min interval.

Average of photocurrent between min 3 and min 7 was used to obtain the dose-response curves (data not shown). Quantitative detection of inactivated *E. coli* O157:H7, *Campylobacter jejuni* and adenovirus were conducted in single experiments with the chambers A to C of the integrated optical-biosensor platform. No fluid leakage was observed during sample processing. Further, the analytical performance of the platform for single pathogen detection is summarized in Table 1. A linear relationship was found between photocurrent and analyte concentration covering at least three orders of magnitude (*R*^2^ > 0.991). Moreover, the minimum pathogen concentration detected by the OPDs was in the range of 10^5^ cells/mL for *E. coli* and *C. jejuni* and 10^−8^ mg/mL for adenovirus. These LODs corresponded to the concentrations equivalent to three times the standard deviation of the baseline levels (*n* = 5). The LOD values for bacteria detection did not differ significantly from those obtained with standard array methods [17] and previously reported chemiluminescence immunoassay schemes [37]. The period for sample incubation (15 min) could be extended to further improve the LOD. However, this can compromise the rapid detection. The precision of detection for all three OPDs was relatively good as relative standard deviation for measuring 5 × 10^5^ cells/mL *E. coli*, 5 × 10^5^ cells/mL *C. jejuni* and 1 × 10^−7^ mg/mL adenovirus was <10%. The method has employed covalent binding of antibody to the PMMA surface. This is highly appropriate for single-use microfluidic devices, ideal to point-of-care applications. Although antibody-to-surface binding of non-covalent nature may reduce the reagents costs [24], the covalent binding is still preferred to obtain better detection reproducibility.

### 3.3. Multiplexed Pathogen Detection

**Figure 5.**
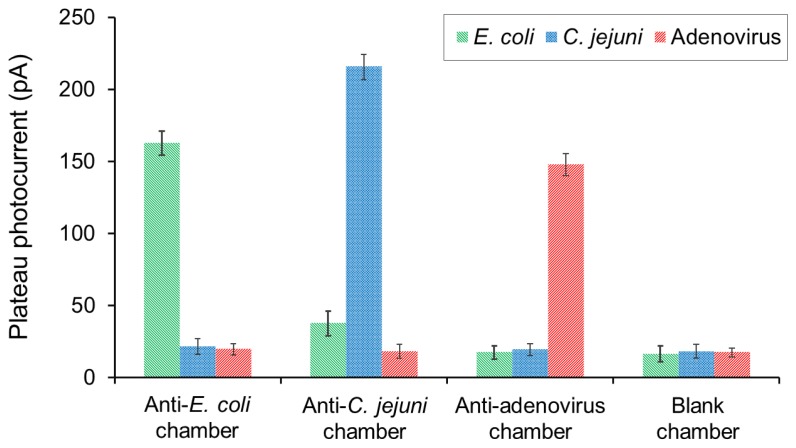
Cross-reactivity tests using single samples of *E. coli*, *C. jejuni* and adenovirus. Detection of each pathogen was tested in four chambers, and the photocurrent measurements were performed in parallel.

In order to enable the multiplexed detection of pathogens in one sample, cross-reactivity events have to be excluded. Tests of antibody-antibody recognition were firstly conducted. For the tests biotinylated antibodies against *E. coli*, *C. jejuni* and adenovirus were loaded into chambers A to D of the integrated platform. None of the biotinylated antibodies interacted with the antibodies immobilized onto the PMMA surface because all measured photocurrents were below the value encountered for the blank chamber. Furthermore, individual samples consisting of 6.5 × 10^6^ cells/mL *E. coli*, 3 × 10^7^ cells/mL *C. jejuni*, 5 × 10^−7^ mg/mL adenovirus were separately incubated within the four chambers. The chemiluminescence signals were recorded in parallel as illustrated in Figure 2. The photocurrent results (Figure 5) showed that the immobilized antibodies presented high specificity for their corresponding targets. Although the detection signal for *E. coli* was slightly higher in the anti-*C. jejuni* chamber comparing to the blank chamber, there was insufficient evidence of cross-reactivity of the immobilized antibodies toward nonspecific pathogens.

**Figure 6.**
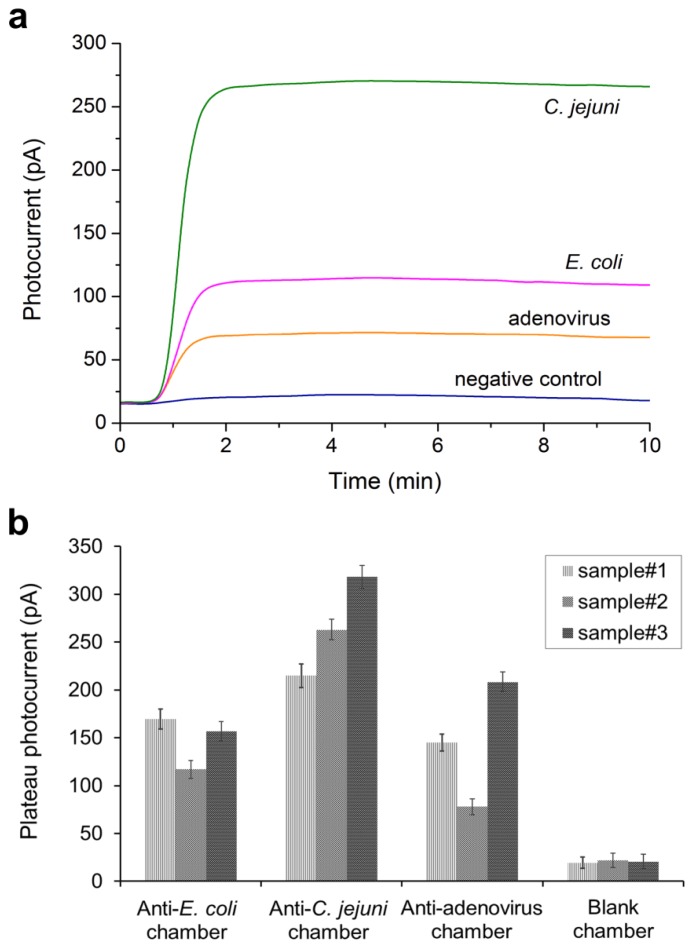
(**a**) Transient chemiluminescence signals for simultaneous detection of *E. coli* O157:H7, *C. jejuni* and adenovirus at one spiked sample. (**b**) Multiplexed detection tests using three mixture samples of the three pathogens (labelled as sample#1, sample#2 and sample#3). Sample#1 contained 6.5 × 10^6^ cells/mL *E. coli*, 3 × 10^7^ cells/mL *C. jejuni* and 5 × 10^−7^ mg/mL adenovirus. Sample#2 was made of 5×10^6^ cells/mL *E. coli*, 5 × 10^7^ cells/mL *C. jejuni* and 1 × 10^−7^ mg/mL adenovirus. Sample#3 contained 6.5 × 10^6^ cells/mL *E. coli*, 1 × 10^8^ cells/mL *C. jejuni* and 5 × 10^−6^ mg/mL adenovirus.

Moreover, the integrated optical-biosensor platform was challenged with mixture samples of all three pathogens. Figure 6a demonstrates the simultaneous detection of 5 × 10^6^ cells/mL *E. coli*, 5 × 10^7^ cells/mL *C. jejuni* and 1 × 10^−7^ mg/mL adenovirus at one water sample. The transient photocurrent responses were recorded from chambers A–D of the integrated device. The average photocurrent taken from the plateau of the transient signal was then plotted for three mixture samples (Figure 6b). The results showed the ability of the device to distinguish different pathogen concentrations in multiplexed conditions at high sensitivity. However, there may be a possibility that varying amounts of pathogen reach the target chamber, as the fluid flow separates after sample loading (Figure 1). This might be the reason that different photocurrents were recorded for the same *E. coli* concentration spiked into two mixture samples (Figure 6b).

The analysis time per multiplexed detection test was 30–35 min, including sample loading, incubation and photocurrent measurement. A test analysis time of 30 min may be sufficient for point-of-care applications [23], and conventional detection methods typically need 1–2 h at least to obtain an accurate result [16]. For instance, a microbiological method consisting of sample culture, biochemical and serological test, which is widely used for routine bacteria detection, can take 3–4 days at best to provide a result [38]. Furthermore, although other chemiluminescence assay techniques showed an overall analysis time of 18 min [37], the multiplexed optical detection was achieved using expensive, non-integrated CCD cameras.

Further multiplexed detection tests were performed by spiking drinking water and surface water samples with inactivated bacteria. The water samples were filtered by 0.45-μm pore size filters prior to bacteria spiking. Similar concentration of *E. coli* and *C. jejuni* was used for all spiked mixture samples. Both *E. coli* and *C. jejuni* were detected in parallel with the photocurrent measured from chambers A and B of integrated platform. To determine recovery rates the platform was previously calibrated using both bacteria separately spiked in drinking water and surface water. The results of recovery are summarized in Table 2. The recovery of all tested samples was between 79% and 107%; however, lower recovery with higher standard deviation was encountered for the spiked surface water samples. This indicated that non-filtered organic and non-organic matter may have interfered with the immunoassays, as described in the literature [39]. Nevertheless, it was shown that the integrated optical-biosensor platform has potential for parallel detection of bacteria in real water samples.

Pre-concentration of bacteria and viruses would be necessary to analyse large volumes of real water samples [21,38]. For this purpose, a micro total analysis system would be realized by incorporating microfiltration and/or immunomagnetic separation into the integrated optical-biosensor platform. Notwithstanding the advantages of a total analysis system, on-chip pre-enrichment of pathogens would lead to longer analysis time and make the manufactured chips more costly. Instead, the design and fabrication of the presented platform would encourage its use as a disposable device. The platform would cost <US$30 after mass production of both OPD array and microfluidic chip.

## 4. Conclusions

The development of an OPD array integrated multi-chamber microfluidic biosensor for multiplexed detection of pathogens was shown. High-responsivity, high-stability OPDs made of poly(2,7-carbazole)/fullerene were employed for monitoring chemiluminescence assays performed onto transparent PMMA microfluidic chip. Simple spin-coating and injection moulding have been used for the fabrication of the OPD array and PMMA chip, enabling mass production of the integrated optical-biosensor platform. This would be interesting for point-of-care applications requiring low-cost monitoring devices. Single pathogen experiments showed LODs of 5 × 10^5^ cells/mL, 1 × 10^5^ cells/mL and 1 × 10^−8^ mg/mL for *E. coli* O157:H7, *C. jejuni* and adenovirus, respectively. High detection precision and specificity for each pathogen and working ranges of at least three orders of magnitude were demonstrated. By combining all three pathogens in one sample the feasibility of the integrated platform to detect multiple bacteria and virus simultaneously was shown. Multiplexed detection was also extended to complex samples. Acceptable recovery rates for bacteria were obtained in spiked drinking water and surface water. One multiplexed detection test may be completed in less than 35 min, although a relatively large volume of expensive chemiluminescence reagents is used for the tests. Nevertheless, rapid detection of up to sixteen analytes can be accomplished with the device, and its further miniaturization is also possible.

## Figures and Tables

**Table 1.** Analytical performance for detection of three pathogens in single experiments.

**Analytical Features**	***E. coli***	***C. jejuni***	**Adenovirus**
**Linear Range**	5 × 10^5^ to 1 × 10^8^ (a)	1 × 10^5^ to 1 × 10^8^ (a)	5 × 10^−8^ to 1 × 10^−5^ (b)
**Limit of Detection**	5 × 10^5^ (a)	1 × 10^5^ (a)	1 × 10^−8^ (b)
**Precision of Detection (c)**	4.6	8.8	3.2

(a) Concentration, cells/mL;

(b) Concentration, mg/mL;

(c) Relative standard deviation (RSD), *n* = 3.

**Table 2.** Recovery of spiked bacteria from filtered water.

**Water Matrix**	**Pathogen (a)**	**Recovery (%)**
**Drinking Water**	*E. coli*	87 ± 14
	*C. jejuni*	107 ± 9
**Surface Water**	*E. coli*	79 ± 23
	*C. jejuni*	102 ± 27

(a) Tested in mixture samples.
